# Near-Field Target Detection with Range–Angle-Coupled Matching Based on Distributed MIMO Radar

**DOI:** 10.3390/s25227003

**Published:** 2025-11-16

**Authors:** Quanrun Cheng, Yuhong Zhang, Cao Zeng, Zhigang Zhou, Guisheng Liao, Haihong Tao

**Affiliations:** 1 National Key Laboratory of Radar Signal Processing, Xidian University, Xi’an 710071, China; chengquanrun@stu.xidian.edu.cn (Q.C.); czeng@mail.xidian.edu.cn (C.Z.); zhigangzhou@stu.xidian.edu.cn (Z.Z.); liaogs@xidian.edu.cn (G.L.); hhtao@xidian.edu.cn (H.T.); 2 School of Electronic Engineering, Xidian University, Xi’an 710071, China

**Keywords:** distributed MIMO radar, FD-LFM signal, near-field target, multi-dimensional matching scheme, GPU

## Abstract

With respect to distributed MIMO radar systems, conventional far-field detection methods fail under near-field conditions due to significant wavefront curvature, which inevitably results in target energy loss and erroneous parameter estimation. To solve this problem, we propose a near-field target detection framework based on range–angle-coupled matching in this study. Firstly, we design the linear frequency modulation by frequency division (FD-LFM) signal. In addition to offering favorable orthogonality and Doppler tolerance, the transmitter of distributed MIMO radar employs a wide beamwidth to mitigate the low scanning efficiency associated with beam positioning in distributed phased array (PA) radar systems. Secondly, we develop a three-dimensional grid-based echo model for near-field targets in range–azimuth–elevation domain. Specifically, we conceive a coherent pulse integration method via multi-dimensional matching, which enables precise delay alignment and echo accumulation across all transmit–receive pairs for accurate near-field target detection. Thirdly, we propose a parallelization scheme for distributed MIMO radar near-field processing. Our proposal not only compensates effectively for spherical wave propagation effects but also achieves real-time processing through GPU acceleration. Finally, our proposed method’s feasibility of high resolution and effectiveness of near-field detection have been verified by field experimental simulation and actual measurement processing results.

## 1. Introduction

In recent years, various studies have been conducted on distributed MIMO radars, prospering the development of radars such as centralized phased array (PA) radar [1,2,3], centralized MIMO radar [4,5], sparse array MIMO radar [6,7,8], bistatic/multi-static radar [9,10,11,12,13], and distributed coherent PA radar [14,15]. Distributed coherent MIMO radar usually has a node-level array element spacing that is typically 100 times greater than its wavelength. As the aperture of the distributed radar increases, the far-field target detection methods may fail [16]. This is because the far-field beamform of the radar only considers the azimuth-pitch 2-dimensional (2D) matching. However, under near-field conditions, the target distance is highly coupled with azimuth and pitch. If far-field methods are used for near-field detection, it will result in the loss of target energy and erroneous parameter estimation. To solve this problem, a range-azimuth-pitch three-dimensional (3D) beamforming is presented to improve the target detection performance under near-field scenarios [17]. However, the existing literature lacks research on GPU acceleration of distributed radar signal processing under near-field conditions. As the production cost of drones and unmanned aerial vehicles (UAVs) continues to plummet, the application of clustered UAVs in civil low-altitude economy (involving but not limited to express delivery by UAV formation and UAV group firefighting) and in clustered UAV cooperation for military purposes has been prospering. With respect to clustered UAVs, discriminating specific individual UAVs with high resolution still remains challenging due to the small range among them [18]. Conventional centralized radar is often limited by antenna aperture (such as installation space and weight constraints), by adopting which discriminating high-resolution clustered targets is rendered difficult. In contrast, distributed radar exhibits an overwhelming advantage in discriminating high-resolution cluster targets due to its ultra-thin distribution of node spacing [19,20,21,22].

As one of the important countermeasures of monitoring UAVs, array radar is capable of performing all-weather, long-range detection duties with high precision [23]. However, the large, dense array radar system required to achieve high spatial resolution has inherent shortcomings such as high system complexity, expensive manufacturing and maintenance cost. Therefore, it is urgent to conduct an in-depth study on the detection technology of sparse-array high-resolution radar, satisfying long-range high-resolution capability while reducing system complexity [24]. Distributed MIMO radar sparsely arranges array elements under the constraints of air, time, and frequency phase reference. Meanwhile, based on unified time-frequency benchmarks, the idea of multi-dimensional joint matching is adopted to improve the resolution capability. In addition, by designing a new scheme and proposing a new method to reduce the side flap of the direction map of the sparse array, it is possible to realize high resolution of multiple targets [25].

Unfortunately, distributed MIMO radar confronts great challenges in multi-target detection [26], one of which is the commonly known near-field target location. Under scenarios of near-field processing mode, the proximity of the target to the radar results in highly nonlinear characteristics of the radar-detected signals, which suggests that conventional far-field processing mode becomes inapplicable [27].

In 2008, the US Air Force Laboratory carried out the field test of the prototype system of Distributed Aperture Radar (DAR), and successfully detected two moving vehicles on the road [28]. To achieve coherent processing across distributed nodes, extremely high-precision synchronization technology is required, which represents one of the most significant technical challenges. Additionally, ultra-high-speed, low-latency data links are necessary for exchanging massive amounts of raw or processed data among nodes. The associated network architectures and processing algorithms are not addressed. In 2020, Liu et al. discussed the idea of cooperative detection adopting distributed MIMO radar [29], and further classified the distributed MIMO radar system according to the antenna array form and signal processing mode. Their modeling of the radar environment depends on idealized assumptions such as perfect knowledge of target states and the absence of electromagnetic (EM) interference. Despite that this approach demonstrates the superior performance of the algorithm, it is highly time-consuming and requires a substantial amount of interaction data.

Due to the coherent synthesis of the emission space of the distributed coherent MIMO radar, the scanning efficiency of the emission wave position is usually reduced to increase its maximum detection range, which hardly improves the capabilities of the detection performance. In this study, we proposed a multidimensional-domain matching signal-level coherent and pulse synthesis method for near-field target detection, which not only elevates coherent integration performance and parameter estimation accuracy, but also reduces processing time without deteriorating its detection reliability. Firstly, a three-dimensional grid echo model of near-field targets based on range-azimuth-elevation was established. Secondly, a phase compensation function was designed to realize accurate delay alignment and coherent integration across all transceiver pairs. Finally, a fully parallelized computation architecture on GPU was implemented to accelerate multi-dimensional matching process, enabling real-time near-field detection.

The key contributions of this study are summarized as follows.

(1) A 3-D discretized echo signal model in range, azimuth, and elevation is established for near-field targets. A coherent pulse integration method via multi-dimensional matching is proposed, enabling precise delay alignment and echo accumulation across all transmit–receive pairs.

(2) The parallelization potential of our proposed multi-dimensional matching method is analyzed across the node, spatial wave position, and slow-time domains. A parallel processing architecture tailored to distributed MIMO radar near-field computation is developed, leveraging CPU + GPU heterogeneous server clusters.

(3) Real-world experiments using distributed radar hardware have validated the effectiveness and high-resolution capability of the proposed method in detecting multiple near-field targets.

The remainder of this paper is organized as follows. Section 2 outlines the related work of the key technologies of distributed MIMO radar. Section 3 presents a system model for distributed MIMO radar. Section 4 highlights the proposed near-field scheme. Section 5 presents and discusses numerical simulations and experimental results. Section 6 summarizes conclusions of this paper.

The following is an introduction to domestic and foreign radar systems with different layouts.

## 2. Related Work

### 2.1. The Technology of Designing Transmitting Waveform

Waveform diversity gives MIMO radar great potential. The waveform parameters that MIMO radar can design and the corresponding processing methods it adopts are much more flexible than those of PA radar, and the waveform design process and signal processing are more complicated than PA radar. The early distributed radar adopted a PA system, and the precise control of transmission delay in time-sharing exhibited the drawbacks of low transmission efficiency. Therefore, it is necessary to study the equivalent approaches of precise regulation of wide-range receiving delay in distributed MIMO systems. In the aspect of transmitting waveform design, various waveform design schemes such as SIAR waveform [7], MIMO frequency division [30], code division [31], time division [32], and DDM [33] have emerged, which expands and deepens the application of radar in various fields. By optimizing the transmission waveform, the signal quality and anti-jamming ability of radar can be improved. In 2021, Bai et al. put forward the multi-pulse joint transmission strategy and sliding-window echo processing algorithm. They analyzed the relationship between related factors, verified their effects on orthogonality improvement, and the relationship between Doppler tolerance and coherence time through numerical simulation, providing a theoretical foundation for subsequent studies. However, its computation load and processing time remain significantly higher than those of conventional single-pulse processing methods. Moreover, the performance evaluation in the paper was primarily conducted under the assumption of zero-Doppler scenarios, leaving its robustness in practical dynamic environments insufficiently explored. Therefore, further in-depth research on environmental adaptability is imperative for its practical deployment [34]. In 2022, Park et al. proposed a multiplexing scheme using M-phase shift keying (MPSK) code, which was successfully realized by using a 4 × 8 MIMO uniform linear array (ULA) antenna through the most advanced FMCW radar. This approach not only solved the speed ambiguity problem in MIMO FMCW radar system but also estimated the accurate 4-D information of the target. However, its primary shortcomings lie in the insufficient discussion of its robustness against practical hardware phase noise and the failure to adequately address the associated practical engineering costs, such as increased computational complexity and elevated hardware requirements [35]. In 2024, Su et al. proposed a phase-coded passive jamming (PCPJ) suppression method based on square nonlinear transform and fractional Fourier transform (FRFT). The algorithm restored the useful signal components shifted by PCPJ modulation and improved the target-to-jamming ratio (TJR). The simulation and experimental results proved the effectiveness of this algorithm. The primary limitation lies in its high computational complexity, which may compromise its viability in real-time radar systems. Furthermore, the simulations reported in the literature predominantly focus on single-target scenarios, and the effectiveness of the reported approach in dense multi-target environment necessitates further validation [36]. In 2024, Bai et al. reported an effective echo processing algorithm and optimization criterion for phase coding set through multi-pulse joint processing. They established an accelerated optimization algorithm based on global and local search to reduce computation complexity. Meanwhile, the orthogonality gain and time advantage were verified by simulation. However, the value of this approach is currently more evident at theoretical level and in offline design. Its most significant limitations lie in its substantial computation complexity and the unresolved uncertainty regarding its adaptability in dynamic target environment [37].

### 2.2. Far-Field and Near-Field Division Technology

Since the aperture of distributed coherent MIMO radar is larger than that of centralized radar, the target is often located in the near field of distributed coherent MIMO radar. Under this circumstance, the far-field plane wave model of conventional centralized radar becomes ineffective, to which the near-field spherical wave model must be considered. The transmitting and receiving angle of distributed coherent MIMO radar differs, which is different from that of centralized radar. Under the near-field spherical wave model of distributed coherent MIMO radar, the target receiving and transmitting delay not only correlates with the transmitting and receiving angle, but also with the transmitting and receiving range. Hence, accurate discrimination between far field and near field is of critical importance to improve the accuracy of radar detection. In 2009, Ke et al. proposed two novel algorithms using fourth-order statistics. The simulation results suggest that conducting DOA and range estimation for near-field narrowband sources provides better performance than traditional approaches [16]. In 2011, Leigsnering et al. reported their broadband near-field radar imaging method based on non-equidistant fast Fourier transform (NFFT) to reduce the computation complexity. The experimental results suggest that the processing speed of this approach has been greatly improved while the error introduced is kept at a very low level [38]. In 2012, Islam et al. established two robust beamforming algorithms based on diagonal loading method, which broadened the idea of far-field beamforming to near-field broadband beamforming, and exhibited the robustness and enhanced performance of the proposed broadband beamformer [39]. In 2012, Liao et al. proposed a robust beamforming algorithm, which applied the convex optimization method to correct the steering vector error of the beam for the worst-case performance of near-field steering vector mismatch, and constrained the algorithm on the source signal to minimize the output variance while maximizing the output SINR. The simulation results of 10-element uniform linear array verified the effectiveness of this proposal [40]. In 2021, Yu et al. proposed an approach to solve the ambiguity of DOA estimation by using double V-shaped arrays with common points. The simulation results suggested that this approach can effectively solve the ambiguity problem of DOA estimation of near-field distributed array broadband signals. The limitations primarily lie in the potentially over-idealized simulation scenarios: experiments are conducted under ideal conditions with high SNR, perfect channel characteristics, and error-free array calibration, while the computation complexity of the corresponding algorithm is excessively high [41]. In 2022, Tian et al. studied a hybrid far-field and near-field location method using a two-stage reduced-order (RARE) estimator. Different from the existing methods, this approach is based on incoherent distributed (ID) source model, which is more applicable for multipath and fast time-varying channels. The simulation results successfully estimated the angle expansion and range expansion. Its primary limitations reside in the high computation complexity and the lack of thorough evaluation regarding its sensitivity to array calibration errors and practical impairments [42]. Besides the Leigsnering et al., which reported a radar imaging method based on non-equidistant fast Fourier transform (NFFT) to reduce the computation complexity, there is a recent imaging model to independently estimate the path lengths from the TX position and RX position to an equivalent reflection point (ERP) on the object surface for precise object reconstruction, which was validated on ray tracing data [43].

### 2.3. Spatial Coherent Accumulation Technique

In spatial coherent accumulation technology, centralized far-field model beamforming and far-field MIMO beamforming have also been deeply studied. However, these approaches still confront many challenges in practical applications, such as anti-interference capability in complicated environments and target detection accuracy [4]. Improving the spatial coherent accumulation technology can enhance the radar performance in detecting and tracking targets. The US Lincoln Laboratories have carried out a lot of studies on the technology of distributed array coherent synthetic radar. In 2004, the Lincoln Lab published an ASTIA Documents (AD) report [44], and published its preliminary research results for the first time. In 2006, it published an article [14] in IEEE, announcing a major breakthrough it had made. Specifically, two broadband radars completely realized coherent transmission and reception, and achieved the maximum gain of 9 dB, which was mentioned twice by Dr. Eli Brookner, a lifetime member of IEEE and a radar expert, in the articles on phased array and radar breakthrough written in 2007 and 2008, respectively [45,46]. In 2012, Gao et al. of the No. 23rd Institute of the Second, Institute of China Aerospace Science and Industry Group adopted two processing methods of receiving phase to receive and transmit coherent signals. They realized the signal-to-noise ratio (SNR) gains of the two radar nodes reached 5.7 dB and 8.5 dB, respectively, which are 0.3 dB and 0.5 dB lower than those of the theoretical values [15]. In 2008, Amuso et al. first reported two robust techniques using spatial diversity distributed aperture to detect clustered super-resolution targets adopting a MIMO radar system, and carried out the field test of a distributed coherent MIMO radar prototype system through the US Air Force laboratory, and successfully detected two moving vehicles on the road [28].

In this study, we propose a multi-dimensional domain matching method of distributed MIMO radar using FD-LFM signal, which was verified by field measurement. Since the FD-LFM signal has the advantages of excellent multi-transmission orthogonality and Doppler tolerance, the research on multi-dimensional matching detection method in this study mainly focuses on FD-LFM signal.

## 3. Problem Description

### 3.1. Signal Model for Distributed MIMO Radar

#### 3.1.1. Near-Field Targets

In airborne radar scenarios, all airspace targets are located within the far-field detection region of the airborne radar, and the corresponding steering vector depends only on the angle between the target direction and the antenna array. As developed and adopted in [47,48], both the signal model and interference model depend heavily on the use of such a steering vector that is closely related to angles.

It must be noted that the above situation becomes increasingly complicated for distributed MIMO ground-based radar. Given a distributed MIMO antenna array with an effective and maximum aperture of *L* and an operating wavelength of λ, a target located in the far-field detection region must satisfy the following three criteria:

(1) r≫L,

(2) r≫λ,

(3) $$r\gg 2L^{2}/\lambda ,$$

For typical distributed aperture values of L=100 m (λ=0.2 m), the far-field region begins at a distance boundary of approximately 100 km. Apparently, both airspace targets and interfering sources are all located in the near-field detection region for arrays with widely distributed radar nodes. Although the concept of steering vector can still be retained, its computation depends on the two-dimensional factors of angle and range, i.e., each grid point in airspace must correspond to its own steering vector. More importantly, the generation of received wideband radar echo and the coherent signal processing over the distributed MIMO array with frequency diversity waveform require true time delays (TTDs), as opposed to the phase shifts used in narrowband radar processing.

#### 3.1.2. Targets Position Coordinates

**Figure 1:**
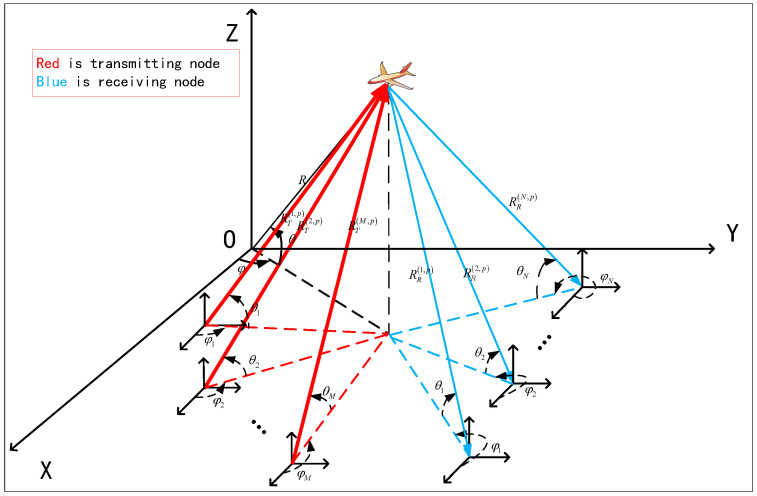
Transmitting and receiving model of distributed MIMO array.

Under the aforementioned scenario, the TTDs must be developed and adopted in distributed MIMO radar system. As shown in Figure 1, distributed MIMO array consists of *M* transmitting nodes and *N* receiving nodes. Within it, the symbols of θ and φ, respectively, represent the elevation angle and azimuth angle of the incoming wave direction of the target relative to the reference point *O*, and *R* represents the Euclidean distance between the target and the reference point. Furthermore, the terms $\theta_{m}^{\left(e,p\right)},\varphi_{m}^{\left(e,p\right)},R_{m}^{\left(e,p\right)}$ denote the elevation angle, azimuth angle, and the range between the *p*-th target and the center of the *m*-th transmitting node, respectively, the terms $\theta_{n}^{\left(r,p\right)},\varphi_{n}^{\left(r,p\right)},R_{n}^{\left(r,p\right)}$ denote the elevation angle, azimuth angle, and the range of the *p*-th target relative to the center line of the *n*-th receiving node, respectively, in the unified array coordinate system.

Within a coherent processing interval (CPI), when the first pulse encounters the *p*-th target, the target position with respect to the reference node can be expressed in three-dimensional (3D) Cartesian coordinates as follows:

(4) $$\left\{\begin{array}{c} x_{p}^{0}=R_{0}^{\left(p\right)}\cos\theta_{0}^{\left(p\right)}\cos\varphi_{0}^{\left(p\right)}\\ y_{p}^{0}=R_{0}^{\left(p\right)}\cos\theta_{0}^{\left(p\right)}\sin\varphi_{0}^{\left(p\right)}\\ z_{p}^{0}=R_{0}^{\left(p\right)}\sin\theta_{0}^{\left(p\right)} \end{array},\right.$$

where $\left(R_{0}^{\left(p\right)},\theta_{0}^{\left(p\right)},\varphi_{0}^{\left(p\right)}\right)$ is the spherical coordinates of target.

#### 3.1.3. TTDs Within a Single Pulse

At the transmitting end of Figure 1, the Euclidean distance between the *m*-th transmitting node and the *p*-th target in the airspace and its corresponding transmitting TTD are, respectively, written as follows:

(5) $$R_{m}^{\left(e,p\right)}=\sqrt{{\left(x_{m}-x_{p}^{0}\right)}^{2}+{\left(y_{m}-y_{p}^{0}\right)}^{2}+{\left(z_{m}-z_{p}^{0}\right)}^{2}},$$

(6) τm(e,p)=Rm(e,p)Rm(e,p)cc,

where $\left(x_{m},y_{m},z_{m}\right)$ is the position coordinate of the *m*-th transmitting node relative to the reference point, *c* denotes the speed of light.

At the receiving end demonstrated in Figure 1, the Euclidean distance between the *p*-th target and the *n*-th receiving node and its corresponding receiving TTD are, respectively, expressed as follows:

(7) $$R_{n}^{\left(r,p\right)}=\sqrt{{\left(x_{n}-x_{p}^{0}\right)}^{2}+{\left(y_{n}-y_{p}^{0}\right)}^{2}+{\left(z_{n}-z_{p}^{0}\right)}^{2}},$$

(8) τn(r,p)=Rn(r,p)Rn(r,p)cc,

where $\left(x_{n},y_{n},z_{n}\right)$ is the position coordinate of the *n*-th receiving node relative to the reference point.

#### 3.1.4. TTDs Within One CPI Duration

During the coherent accumulation time, there are *K* transmit pulses included. At time *k*-th (k=1,2,⋯,K), the 3D velocity of the *p*-th target, denoted as $v^{\left(p,k\right)}$, can be expressed as follows:

(9) $$v^{\left(p,k\right)}=\sqrt{{\left(v_{x}^{\left(p,k\right)}\right)}^{2}+{\left(v_{y}^{\left(p,k\right)}\right)}^{2}+{\left(v_{z}^{\left(p,k\right)}\right)}^{2}}.$$

Assuming that the target is moving at a uniform speed within 100 ms of one CPI duration, this suggests that the corresponding target velocity and direction in each dimension remain unchanged. Therefore, when the *k*-th transmitting pulse within one CPI duration reaches the airspace target, its 3D Cartesian coordinates can be written as follows:

(10) $$\left\{\begin{array}{c} x_{p}^{k}=x_{p}^{0}-\left(k-1\right)T_{r}v_{x}^{\left(p,k\right)}\\ y_{p}^{k}=y_{p}^{0}-\left(k-1\right)T_{r}v_{y}^{\left(p,k\right)}\\ z_{p}^{k}=z_{p}^{0}-\left(k-1\right)T_{r}v_{z}^{\left(p,k\right)} \end{array},\right.$$

where Tr=11fPRFfPRF denotes the pulse repetition time (PRT).

The Euclidean distances between the *p*-th target and the *m*-th transmitting node and the *n*-th receiving node at the *k*-th pulse are, respectively, expressed as follows:

(11) $$R_{m}^{\left(e,p,k\right)}=\sqrt{{\left(x_{m}-x_{p}^{k}\right)}^{2}+{\left(y_{m}-y_{p}^{k}\right)}^{2}+{\left(z_{m}-z_{p}^{k}\right)}^{2}},$$

(12) $$R_{n}^{\left(r,p,k\right)}=\sqrt{{\left(x_{n}-x_{p}^{k}\right)}^{2}+{\left(y_{n}-y_{p}^{k}\right)}^{2}+{\left(z_{n}-z_{p}^{k}\right)}^{2}}.$$

Thus, for the *k*-th (k=1,2,…,K) pulse within one CPI duration, the TTDs are, respectively, expressed as follows:

(13) τm(e,p,k)=Rm(e,p,k)Rm(e,p,k)cc,τn(r,p,k)=Rn(r,p,k)Rn(r,p,k)cc.

### 3.2. Multi-Dimensional Signal Model

#### 3.2.1. FD-LFM Transmitting Signal Model

At the transmitting end of the distributed MIMO radar, the *M* LFM signals with different starting frequencies $f_{m}$ (i.e., FD-LFM waveform) are transmitted simultaneously, which can be represented as follows:

(14) $$s_{m\_IF}\left(t\right)=\mathrm{rect}\left(\frac{t}{T_{p}}\right)\cdot e^{j2\pi f_{m}t}\cdot e^{j\pi \mu t^{2}},m=1,2,\cdots ,M,$$

where $\mathrm{rect}\left(\frac{t}{T_{p}}\right)=\left\{\begin{array}{c} 1,0<t<T_{p}\\ 0,\mathrm{else} \end{array}\right.$, $T_{p}$ is the pulse width, *B* denotes the signal bandwidth, μ=B/Tp is the frequency modulation slope, *f_m_* = (*m* − 1)Δ*f* is the waveform of the *m*-th transmitting node (excluding carrier frequency), where Δ*f* is the frequency interval between the transmitted signals.

**Figure 2:**
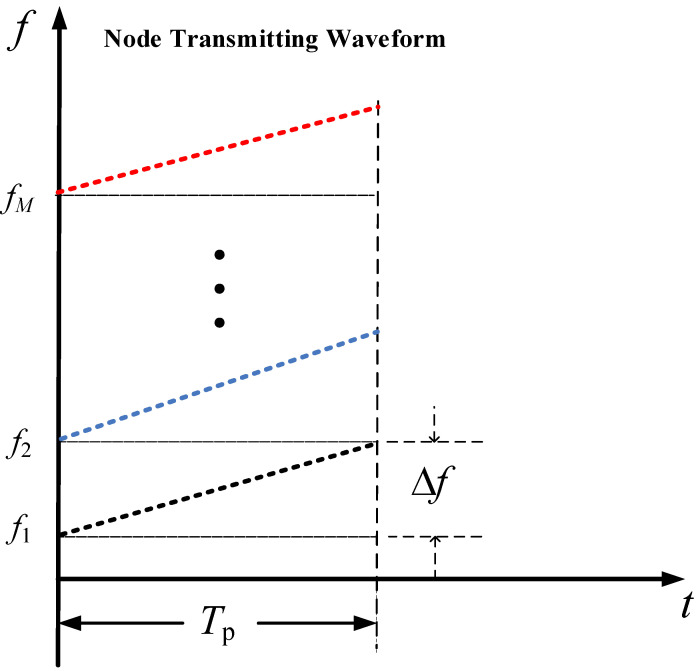
Schematic diagram of time-frequency relationship of FD-LFM signal.

To clearly illustrate the FD-LFM transmitting waveform of the distributed MIMO radar in this study, a schematic diagram of the time–frequency relationship at each transmitting node is plotted in Figure 2. Obviously, it can be noticed that each node has the same parameters of $T_{p}$ and *B*, for which reason the value of μ for each transmitting node is the same. Note that in order to avoid the spectral aliasing problem of the transmitting signal in this study, the frequency interval is set to be equal to the signal bandwidth, i.e., Δf=B.

After up-conversion, the transmitted RF signal at the *m*-th transmitting node for Equation (14) can be further represented as follows:

(15) $$s_{m\_RF}\left(t\right)=\mathrm{rect}\left(\frac{t}{T_{p}}\right)\cdot e^{j2\pi (f_{c}+f_{m})t}\cdot e^{j\pi \mu t^{2}},$$

where $f_{c}$ denotes the carrier frequency. Beyond the formulation presented in Equation (15), it is noteworthy that several recently developed chirp waveforms—distinct from conventional linear frequency modulation (LFM)—are capable of embedding communication information while preserving chirping characteristics. One prominent example is the dual-mode time-domain multiplexed chirp spread spectrum (DM-TDM-CSS), which retains the favorable properties of LFM waveforms while simultaneously embedding communication data [43].

#### 3.2.2. Receiving Echo Signal Model

Since the designed waveforms of *M* LFM signals with different starting frequencies are simultaneously transmitted, the signal expression for reaching the *p*-th target can be expressed as follows:

(16) $$\begin{array}{cc} s_{m}^{\left(e,p\right)}\left(t\right)= & \sum_{m=1}^{M}A_{m}^{\left(e,p\right)}\mathrm{rect}\left(\frac{t-\tau_{m}^{\left(e,p\right)}}{T_{p}}\right)\cdot e^{j\pi \mu {\left(t-\tau_{m}^{\left(e,p\right)}\right)}^{2}}\\ & \cdot e^{j2\pi \left(f_{c}+f_{m}\right)\left(t-\tau_{m}^{\left(e,p\right)}\right)} \end{array},$$

where $A_{m}^{\left(e,p\right)}$ is the signal amplitude at which the *m*-th transmitted signal reaches the *p*-th target. Then, the radar echo signal scattered from the *p*-th target back to the *n*-th receiving node can be written as follows:

(17) $$s_{n}^{\left(r,p\right)}\left(t\right)=s_{m}^{\left(e,p\right)}\left(t-\tau_{n}^{\left(r,p\right)}\right)A_{n}^{\left(r,p\right)},$$

where $A_{n}^{\left(r,p\right)}$ is the signal amplitude from the *p*-th target to the *n*-th receiving node. More specifically, by substituting Equation (16) into Equation (17), the received echo signal of the *n*-th node from the *p*-th target can be expressed as follows:

(18) $$\begin{array}{cc} s_{n}^{\left(r,p\right)}\left(t\right) & =A_{n}^{\left(r,p\right)}\sum_{m=1}^{M}A_{m}^{\left(e,p\right)}\mathrm{rect}\left(\frac{t-\tau_{m}^{\left(e,p\right)}-\tau_{n}^{\left(r,p\right)}}{T_{p}}\right)\\ & \cdot e^{j2\pi \left(f_{c}+f_{m}\right)\left(t-\tau_{m}^{\left(e,p\right)}-\tau_{n}^{\left(r,p\right)}\right)}\cdot e^{j\pi \mu {\left(t-\tau_{m}^{\left(e,p\right)}-\tau_{n}^{\left(r,p\right)}\right)}^{2}}, \end{array}$$

Subsequently, by performing digital down-conversion operation with respect to the received target echo in Equation (18), i.e., multiplied by $e^{-j2\pi f_{c}t}$, the received baseband echo signal of the *p*-th target can be re-written as follows:

(19) $$\begin{array}{cc} s_{n}^{\left(r,p\right)}\left(t\right) & =\sum_{m=1}^{M}A_{mn}^{\left(p\right)}\mathrm{rect}\left(\frac{t-\tau_{mn}^{\left(p\right)}}{T_{p}}\right)\cdot e^{-j2\pi \left(f_{c}+f_{m}\right)\tau_{mn}^{\left(p\right)}}\\ & \cdot e^{j2\pi f_{m}t}\cdot e^{j\pi \mu {\left(t-\tau_{mn}^{\left(p\right)}\right)}^{2}} \end{array},$$

where the term $\tau_{mn}^{\left(p\right)}=\tau_{m}^{\left(e,p\right)}+\tau_{n}^{\left(r,p\right)}$ denotes the total TTD corresponding to the path to which the *m*-th node reaches the *p*-th target and reflects back to the *n*-th node, and the term $A_{mn}^{\left(p\right)}=A_{m}^{\left(e,p\right)}A_{n}^{\left(r,p\right)}$ denotes the total amplitude of the echo signal for this transmit–receive path.

## 4. Scheme Design of Multi-Dimensional Matching Processing for Distributed MIMO Radar

**Figure 3:**
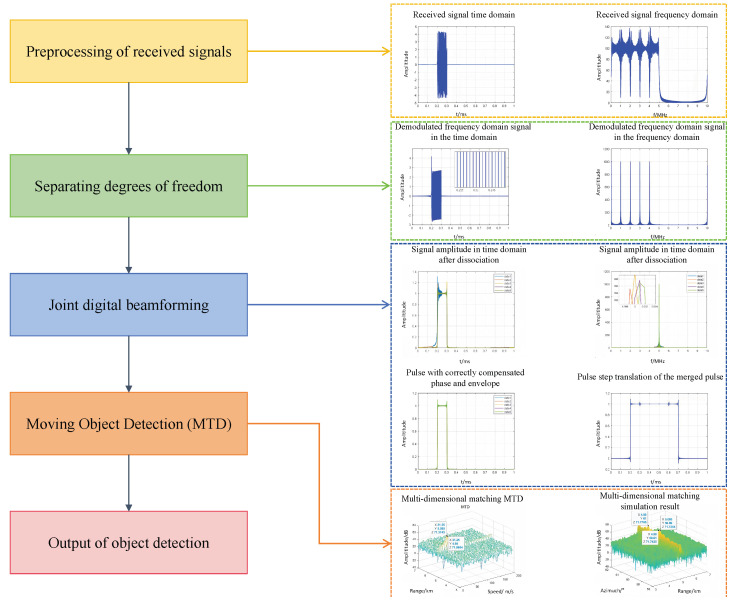
Flowchart of the proposed core process of distributed MIMO radar.

Distributed radar has the advantages of high spatial resolution and strong survivability brought by large aperture. Due to the range–angle coupling in near-field precise matching processing, the computational complexity of distributed aperture radar is huge. Therefore, aiming at solving the problems of high algorithm complexity and large amounts of computation, we studied parallel design based on GPU. As shown in Figure 3, the proposed algorithm consists of three main procedures: separating degrees of freedom, joint digital beamforming, and moving object detection.

### 4.1. Transmitted Signal Separation at the Receiving End

For each receiving node of distributed MIMO radar in Figure 1, the received baseband echo signal can be effectively separated into *M* LFMs with different starting frequencies transmitted by the transmitting nodes using a specially designed matched filter.

Due to the orthogonality of the FD-LFM signal waveform, the matched filtering process equivalent to autocorrelation processing can usually be used to separate the transmitted signals for MIMO radar systems. Meanwhile, the number of corresponding matched filters is consistent with the number of transmitted signals.

#### 4.1.1. Frequency–Domain Representation of the Received Baseband Echo Signal

The frequency–domain expression for the *m*-th transmitted node separated from the received baseband echo signal of Equation (19) can be formulated as follows:

(20) $${\hat{S}}_{n}^{\left(m,p\right)}\left(f\right)=s_{n}^{\left(r,p\right)}\left(t\right)e^{-j2\pi f_{m}t},m=1,2,\ldots ,M,$$

For subsequent deduction, the frequency–domain echo signal in Equation (20) is decomposed into the superposition of the baseband echo of the *m*-th transmitted signal and the baseband signals from the remaining M−1 transmitted signals. Also, a digital down-conversion operation is performed. As a result, Equation (20) can be further rewritten as follows:

(21) $$\begin{array}{c} \begin{array}{c} {\hat{S}}_{n}^{\left(m,p\right)}\left(f\right)=A_{mn}^{\left(p\right)}\mathrm{rect}\left(\frac{t-\tau_{mn}^{\left(p\right)}}{T_{p}}\right)\cdot e^{-j2\pi \left(f_{c}+f_{m}\right)\tau_{mn}^{\left(p\right)}}\\ \cdot e^{j\pi \mu {\left(t-\tau_{mn}^{\left(p\right)}\right)}^{2}}+\sum_{j=1,j\neq m}^{M}\Omega \end{array} \end{array},$$

where an expression of $\Omega =A_{jn}^{\left(p\right)}\mathrm{rect}\left(\frac{t-\tau_{jn}^{\left(p\right)}}{T_{p}}\right)\cdot e^{-j2\pi \left(f_{c}+f_{j}\right)\tau_{jn}^{\left(p\right)}}\cdot e^{j2\pi (f_{j}-f_{m})t}\cdot e^{j\pi \mu {\left(t-\tau_{jn}^{\left(p\right)}\right)}^{2}}$ is introduced here for simplicity.

#### 4.1.2. Frequency–Domain Representation of the Separated Transmitter Signals

In order to achieve the frequency–domain matching filtering process derived later, a prior task of the frequency–domain expression for the *m*-th separated transmitted signal must be explored using the principle of stationary phase (POSP) [49,50]. Then, further representation can be solved through the Fresnel integration [51]. For the POSP involved here, it can transform the original complex integral into an integral computation that solves for the neighborhood of the stationary point to obtain an approximate solution to the original integral.

As derived in Equation (19), the frequency–domain expression of this complex echo result can be expressed as follows:

(22) $$S_{n}^{\left(p\right)}\left(f\right)=s_{n}^{\left(r,p\right)}\left(t\right)e^{-j2\pi ft}dt,$$

By further extracting the phase φ(t) that changes over time, the above Equation (22) can be formulated as follows:

(23) $$\varphi \left(t\right)=-2\pi ft+\pi \mu {\left(t-\tau_{mn}^{\left(p\right)}\right)}^{2}-2\pi \left(f_{c}+f_{m}\right)\tau_{mn}^{\left(p\right)}.$$

The derivative of Equation (23) with respect to *t* can be calculated by the following:

(24) $$\frac{d\varphi (t)}{dt}=-2\pi f+2\pi \mu \left(t-\tau_{mn}^{\left(p\right)}\right).$$

As stated in POSP, the stationary phase point is the point where the phase change occurs slowest, and the derivative at this point is 0. Therefore, performing the derivative operation on Equation (24) with dφ(t)dφ(t)dtdt=0 yields the stationary phase point of t*=τmn(p)+ffμμ. Then, Equation (22) can be further converted into the following:

(25) $$S_{n}^{\left(p\right)}\left(f\right)=\frac{A_{mn}^{\left(p\right)}}{\mu }\mathrm{rect}\left(\frac{f}{B}\right)\cdot e^{-j2\pi \left(f+f_{c}+f_{m}\right)\tau_{mn}^{\left(p\right)}}\cdot e^{-j\pi \frac{f^{2}}{\mu }}\cdot e^{j\frac{\pi }{4}}.$$

#### 4.1.3. Frequency-Domain Representation of the Reference Signal

According to Equation (14), the time-domain transmitted signal of the *m*-th node can be specifically written as follows:

(26) $$s_{m}^{\left(e,p\right)}\left(t\right)=\mathrm{rect}\left(\frac{t}{T_{p}}\right)e^{j2\pi f_{m}t}e^{j\pi \mu t^{2}},$$

to which conducting digital down-conversion yields the following:

(27) $${\hat{s}}_{m}^{\left(m,p\right)}\left(t\right)=s_{m}^{\left(e,p\right)}\left(t\right)e^{-j2\pi f_{m}t}.$$

To obtain the reference signal to be used later in the matching filter process, one should start with the computation of the frequency–domain expression for the *m*-th transmitted signal. A derivation of the frequency–domain representation of the *m*-th node, similar to the frequency–domain representation process of the separated transmitter signals from Equation (23) to Equation (25), has been provided in Appendix A. Based on this, the frequency–domain expression of the reference signal experiencing the conjugate operation can be represented as follows:

(28) $$S_{m}^{*(e)}\left(f\right)=\mathrm{rect}\left(\frac{f}{B}\right)e^{j\pi \frac{f^{2}}{\mu }}e^{-j\frac{\pi }{4}}.$$

#### 4.1.4. Frequency–Domain Matched Filter Processing

Generally, matched filtering processing methods can be classified into two categories: frequency–domain processing and time-domain processing approaches. Time-domain matched filtering involves convolving the separated transmitted signal with the conjugate-inverted reference signal [52,53], while the frequency–domain matched filtering transforms the time-domain convolution operation into the frequency domain using fast Fourier transform (FFT), performs multiplication, and then converts back to the time domain through inverse fast Fourier transform (IFFT). Although both methods yield identical results, the frequency–domain approach exhibits superior computational efficiency when processing received echo signals with numerous sampling points [54].

**Figure 4:**
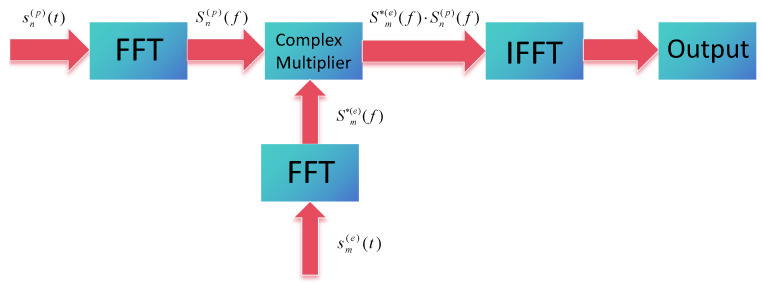
The processing of frequency–domain matched filtering.

**Figure 5:**
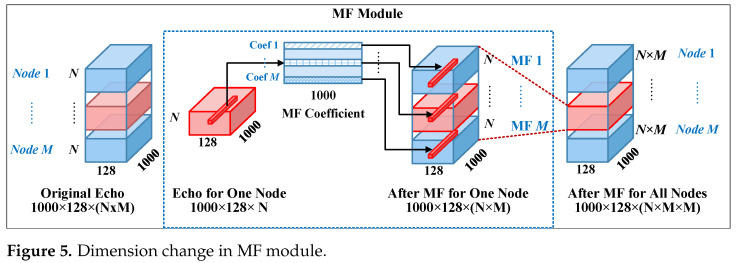
Dimension change in MF module.

For the multi-node MIMO radar system, the frequency matching filtering module can be accelerated using GPUs. For each node, this process for each node can be conceptualized as a dot-product operation between individual pulses from each array element and the matched filter coefficients. As illustrated in Figure 4, it is iteratively performed until all received data from all nodes are processed. Regarding the detailed implementation of transmitted signal separation at the receiving end, a processing flowchart is plotted and presented in Figure 5. It can be observed that the process involves multiplying the echo signal with its corresponding reference signal in frequency domain, followed by an IFFT operation to obtain the time-domain representation of the transmitted signal.

Consequently, the output of the frequency–domain matching process can be mathematically expressed as follows:

(29) $$s_{0}\left(t\right)=\mathrm{IFFT}\left\{S_{m}^{*(e)}\left(f\right)\cdot S_{n}^{\left(p\right)}\left(f\right)\right\},$$

where $S_{m}^{*(e)}\left(f\right)=\mathrm{rect}\left(\frac{f}{B}\right)e^{j\pi \frac{f^{2}}{\mu }}e^{-j\frac{\pi }{4}}$, $S_{n}^{\left(p\right)}\left(f\right)=\frac{A_{mn}^{\left(p\right)}}{\mu }\mathrm{rect}\left(\frac{f}{B}\right)e^{-j2\pi \left(f+f_{c}\right)\tau_{mn}^{\left(p\right)}}e^{-j\pi \frac{f^{2}}{\mu }}e^{j\frac{\pi }{4}}$. The TTD $\tau_{0}$ is given by τ0=2R0(p)2R0(p)cc, where $R_{0}^{\left(p\right)}$ represents the distance from the *p*-th target to the reference point.

For the *k*-th pulse, the frequency–domain multiplication result for Equation (29) can explicitly expressed as follows:

(30) $$\begin{array}{cc} S_{mn}^{\left(MF\right)}\left(f\right) & =S_{n}^{\left(p\right)}\left(f\right)\cdot S_{m}^{*(e)}\left(f\right)\\ & =\frac{A_{mn}^{\left(p\right)}}{\mu }\mathrm{rect}\left(\frac{f}{B}\right)e^{-j2\pi \left(f+f_{c}+f_{m}\right)\tau_{mn}^{\left(p\right)}} \end{array},$$

and the corresponding time-domain expression is further derived as follows:

(31) $$\begin{array}{cc} s_{mn}^{\left(MF\right)}\left(t\right) & =\\ & \frac{A_{mn}^{\left(p\right)}}{\mu }\int_{-\frac{B}{2}+f_{m}}^{\frac{B}{2}+f_{m}}\mathrm{rect}\left(\frac{f}{B}\right)e^{-j2\pi \left(f+f_{c}+f_{m}\right)\tau_{mn}^{\left(p,k\right)}}e^{j2\pi ft}df\\ & =A_{mn}^{\left(p\right)}T_{p}\frac{\sin\left[\pi B(t-\tau_{mn}^{\left(p,k\right)})\right]}{\pi B(t-\tau_{mn}^{\left(p,k\right)})}e^{-j2\pi \left(f_{c}+f_{m}\right)\tau_{mn}^{\left(p,k\right)}} \end{array}$$

where τmn(p,k)=Rm(e,p,k)+Rn(r,p,k)Rm(e,p,k)+Rn(r,p,k)cc denotes the total TTD from the *m*-th transmitting node to the *n*-th receiving node. Specifically, the term $R_{m}^{\left(e,p,k\right)}$ is the transmission path from which the signal transmitted by the *m*-th node reaches the *p*-th target at the *k*-th pulse, while $R_{n}^{\left(r,p,k\right)}$ is the receiving path from the *p*-th target to the *n*-th node at the *k*-th pulse.

As the above observation demonstrates, performing frequency–domain matched filtering at the receiving end achieves an efficient separation of the transmitted signals, separating a total of M×N signals.

### 4.2. Design of the Compensation Function

As shown in Equation (31), it can be noticed that the accumulated gain of coherent synthesis processing at the receiving end of the distributed MIMO radar mainly depends on whether the TTD from the target to each channel can be accurately compensated. To achieve high resolution of airspace targets, it is urgent to finely divide the airspace detection into 3D (range-elevation-azimuth) grids.

#### 4.2.1. 3D Grid Division of Airspace Detection

**Figure 6:**
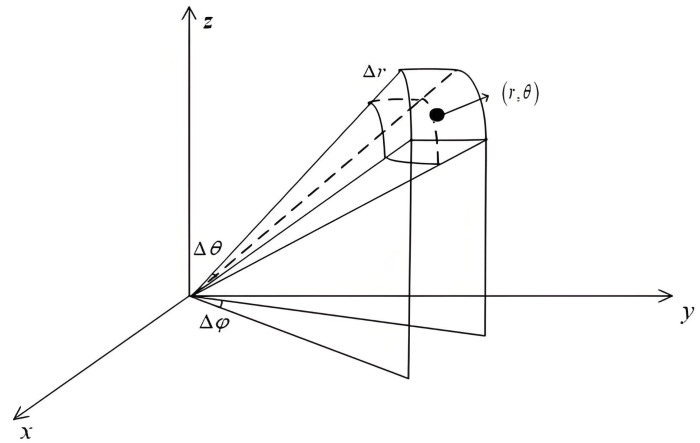
Schematic diagram of a 3D grid resolution unit in airspace detection.

As shown in Figure 6, the airspace detection is divided into a large number of 3D grid points related to the basic resolution units of range–azimuth–elevation; a schematic diagram of a 3D grid resolution unit within it is also depicted here. Typically, the TTDs between the center position of each 3D resolution unit and the array elements can be pre-calculated and stored for compensating the received array echo signals.

Actually, the near-field 3D grid matching processing is a 3D search matching filter process, which requires matching filter processing for each resolution unit in the airspace until the preset target is accurately detected. Therefore, the TTDs at the target location are unknown before the target is detected.

Based on the above principle, for the 3D matched filtering of the current grid point, the TTDs of $\tau_{m}^{\left(e,p,k\right)}$ and $\tau_{n}^{\left(r,p,k\right)}$ in Equation (13) are required to be pre-calculated first, and a compensation function is then further designed to align the matching peak for the transmit–receive pair of mn. Therefore, the amplitude gain of the matching output is maximized only when the target is exactly located at the current grid point. It must be noted that during the matching search process, the search step for distance grids is typically set to half the distance resolution (i.e., Δr=c/4B), while the search step for angles is set to one-tenth of the half-power beamwidth (HPBW) (i.e., $\Delta \theta =\theta_{0.5}/10,\Delta \varphi =\varphi_{0.5}/10$, where $\theta_{0.5}$ and $\varphi_{0.5}$ are the HPBW values for the azimuth and elevation dimensions, respectively).

#### 4.2.2. Accurate Compensation Based on the 3D Grid Resolution Units

For the first pulse within the CPI, the phase compensation function can be formulated as follows:

(32) $$H\left(f\right)=e^{j2\pi \left(f+f_{c}+f_{m}\right)\tau_{mn}^{\left(p,1\right)}}e^{-j2\pi \left(f+f_{c}+f_{m}\right)\tau_{0}^{\left(p,1\right)}},$$

and the corresponding compensation result obtained after frequency–domain compensation in Equation (30) is as follows:

(33) $$\begin{array}{c} S_{mn}^{\left(MF\right)}\left(f\right)H\left(f\right)=\frac{A_{mn}^{\left(p\right)}}{\mu }\mathrm{rect}\left(\frac{f}{B}\right)\cdot e^{-j2\pi (f+f_{c}+f_{m})}\cdot e^{-j2\pi [\tau_{0}^{\left(p,1\right)}+\tau_{mn}^{\left(p,k\right)}-\tau_{mn}^{\left(p,1\right)}]}\\ =\frac{A_{mn}^{\left(p\right)}}{\mu }\mathrm{rect}\left(\frac{f}{B}\right)\cdot e^{-j2\pi \left(f+f_{c}+f_{m}\right)\tau_{0}^{\left(p,k\right)}}. \end{array}$$

The *p*-th target does not cross the grid at the coherent accumulation time as follows:

(34) $$\tau_{0}^{p}=\tau_{0}^{\left(p,1\right)}\approx \tau_{0}^{\left(p,2\right)}\approx \cdots \tau_{0}^{\left(p,K\right)},$$

The target motion during *k* coherent accumulation pulses can be regarded as a straight and uniform motion as follows:

(35) $$v_{r}^{\left(p,k\right)}=v^{\left(p,k\right)}\cos\varphi_{v},$$

where $\varphi_{v}$ denotes the angle between the radial velocity and the real velocity.

(36) $$\tau_{mn}^{\left(p,k\right)}=\tau_{mn}^{\left(p,1\right)}+v_{r}^{\left(p,k\right)}\left(k-1\right)T_{r},$$

When vr(p,k)(k−1)Tr<cc2Fs2Fs, the pulse compression peaks are aligned at the same range cell.

(37) $$s_{mn}^{\left(MF\right)}\left(t\right)\approx A_{mn}^{\left(p\right)}T_{p}\frac{\sin\left[\pi B(t-\tau_{0}^{\left(p,1\right)})\right]}{\pi B(t-\tau_{0}^{\left(p,1\right)})}e^{-j2\pi \left(f_{c}+f_{m}\right)\tau_{0}^{\left(p,k\right)}}$$

### 4.3. Joint Digital Beamforming


(38) $$\begin{array}{cc} s^{\left(DBF\right)}\left(t,k\right) & =\sum_{p=1}^{P}\left(\sum_{n=1}^{N}\sum_{m=1}^{M}s_{mn}^{\left(MF\right)}\left(t\right)+B^{\left(NMF\right)}\right)\\ & =\sum_{p=1}^{P}(T_{p}\frac{\sin\left[\pi B(t-\tau_{0}^{\left(p,1\right)})\right]}{\pi B(t-\tau_{0}^{\left(p,1\right)})}\\ & e^{-j2\pi \left(f_{c}+f_{m}\right)\tau_{0}^{\left(p,k\right)}}\sum_{n=1}^{N}\sum_{m=1}^{M}A_{mn}^{\left(p\right)}\\ & +B^{\left(NMF\right)}) \end{array}$$

### 4.4. Moving Target Detection

Moving target detection (MTD) is a filter bank of multiple band-pass filters, which is commonly implemented using data rearrangement and FFT operations. After the MF module, the fast-time data are sequential in memory to accelerate data access efficiency. Therefore, the data should be rearranged. Then, the FFT operation is applied to transform the data into the Doppler domain, which can be described by the following:

(39) $$\begin{array}{cc} \tau_{0}^{\left(p,k\right)} & =\tau_{0}^{\left(p,1\right)}+v_{r}^{\left(p,k\right)}\left(k-1\right)T_{r} \end{array}$$

Perform FFT on slow time along the same range cell edge as follows:

(40) $$\begin{array}{cc} s^{\left(MTD\right)}\left(t,f_{d}\right) & =\mathrm{FFT}(s^{\left(DBF\right)}\left(t,k\right)) \end{array}$$

then

(41) $$\begin{array}{cc} s^{\left(MTD\right)}\left(t,f_{d}\right) & =\sum_{p=1}^{P}(T_{p}\frac{\sin\left[\pi B(t-\tau_{0}^{\left(p,1\right)})\right]}{\pi B(t-\tau_{0}^{\left(p,1\right)})}\\ & \frac{\sin\left[\pi kT_{r}\left(f_{d}-\frac{2v_{r}}{\lambda }\right)\right]}{\pi kT_{r}\left(f_{d}-\frac{2v_{r}}{\lambda }\right)}\\ & e^{-j2\pi \left(f_{c}+f_{m}\right)\tau_{0}^{\left(p,k\right)}}\sum_{n=1}^{N}\sum_{m=1}^{M}A_{mn}^{\left(p\right)}\\ & +B^{\left(NMF\right)}) \end{array}$$

## 5. Numerical Simulations and Experimental Results

The steps of conducting FD-LFM near-field multi-dimensional matching are deduced above, and the expression of the matching filter function is presented. Hereinafter, the above algorithm will be simulated and verified by setting a specific detection scenario. Firstly, the near-field echo model is used to generate FD-LFM array signal echo, then frequency–domain near-field multidimensional matching is used to process it. Finally, the matching output response is depicted and compared with the set value.

### 5.1. Numerical Simulation of Near-Field Multi-Dimensional Matching Processing Scheme

#### 5.1.1. Simulation Setting

**Figure 7:**
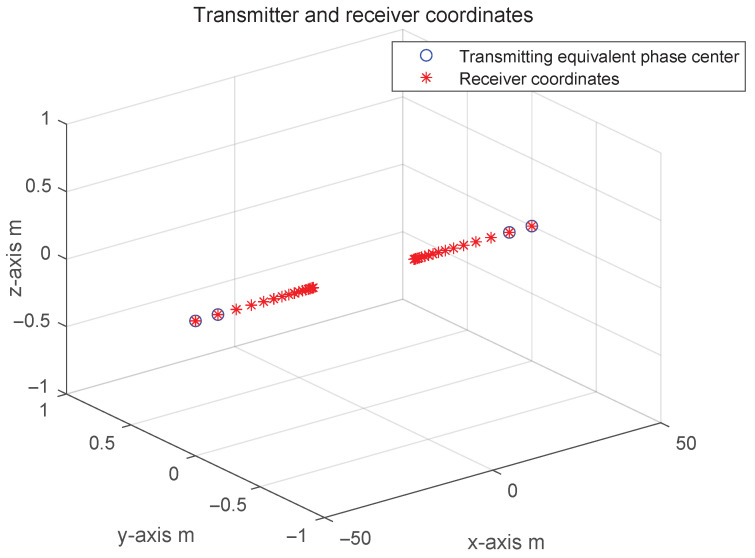
Schematic diagram of the geometry of the sensor array in airspace detection.

In this subsection, the simulation verification of the proposed near-field multi-dimensional matching scheme of distributed MIMO radar is considered and examined for single-objective and multi-objective scenarios. In the simulation, the transmitting nodes and receiving nodes adopt a symmetric exponential distribution linear array (see Figure 7, with the maximum aperture of the array being 100 m). More detailed, the radar parameters and target parameters are, respectively, listed in Table 1 and Table 2. This simulation represents a large-aperture scenario where near-field effects dominate.

#### 5.1.2. Simulation Analysis

**Figure 8:**
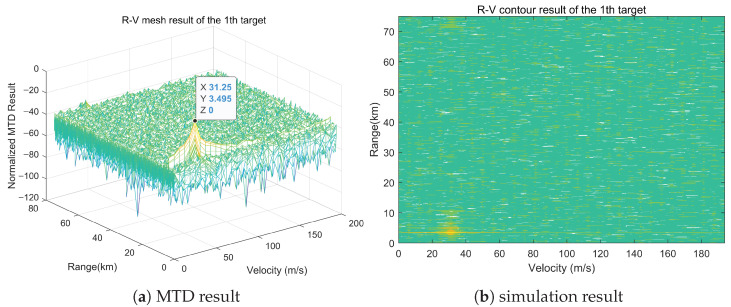
Multi-dimensional matching result at (3.495, 60.01, 31.25).

**Figure 9:**
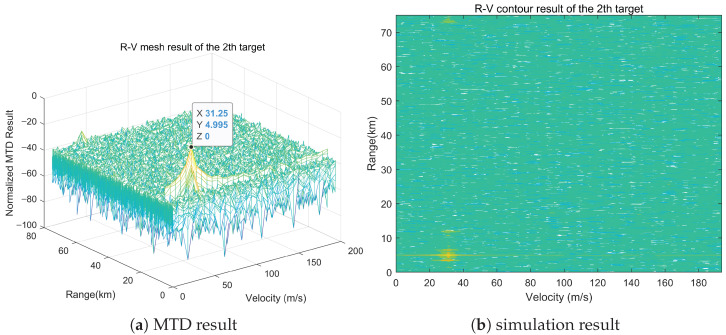
Multi-dimensional matching result at (4.495, 59.9, 31.25).

**Figure 10:**
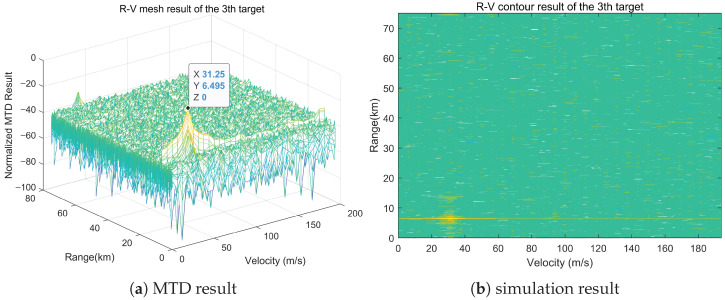
Multi-dimensional matching result at (6.495, 61, 31.25).

It can be observed from Figure 8, Figure 9 and Figure 10 that significant peaks are accumulated at (3.495, 60.01, 31.25), (4.495, 59.9, 31.25), and (6.495, 61, 31.25) after processing the echo signal. The differences between the peak values of the simulation results and the set target parameters in terms of range, azimuth, and Doppler information are within the error range. This proves that the matching method has the capability of high spatial resolution and can effectively discriminate multiple targets with small positional differences in spatial domain.

### 5.2. Experimental Results and Analysis

**Figure 11:**
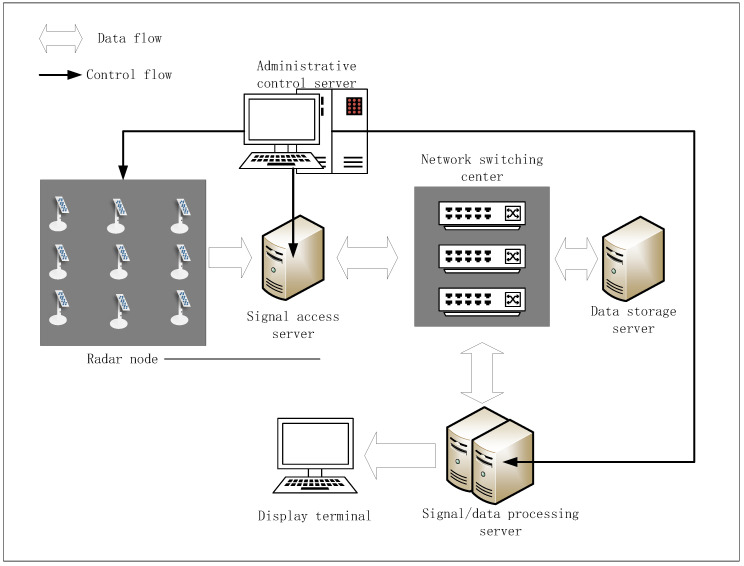
Composition of distributed MIMO radar system.

As shown in Figure 11, the distributed MIMO radar collaborative detection test system mainly consists of radar nodes, management control servers, signal access servers, data storage servers, signal/data processing servers, network switching centers, display terminals, and other devices. A single node usually consists of multiple antenna array elements, in which each node can be configured to receive and operate in single-shot working states. By using the system management software in the management control server, the gigabit switches can manage, schedule, and plan radar nodes, signal/data processing servers, and other devices. The RF signal is processed by demodulation sampling at the node receiving end, and then transmitted to the signal/data processing server through a high-speed switch and optical fiber network cable. The signal/data processing server performs coherent processing, parameter estimation, and track generation on the received echo data, and displays the output in real time on the display terminal.

**Figure 12:**
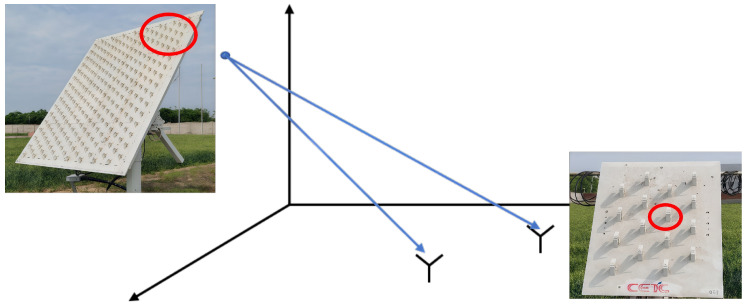
Physical image of distributed MIMO radar nodes.

**Figure 13:**
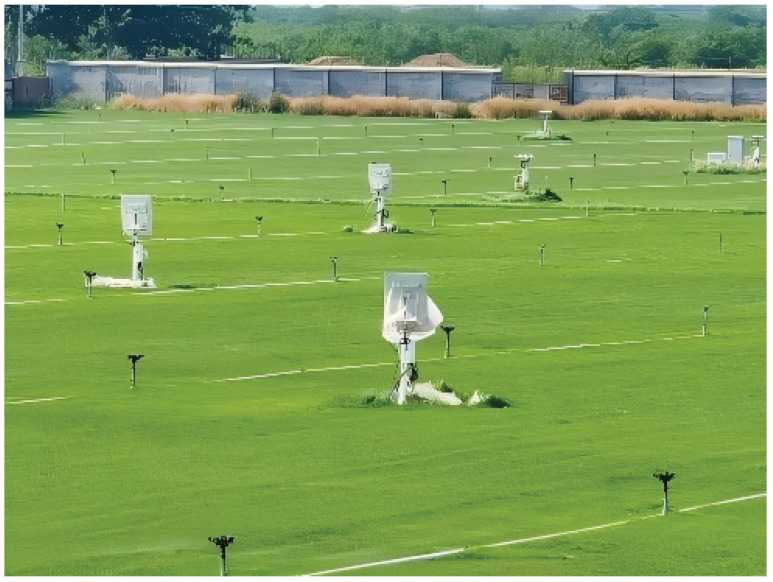
Experiment layout of the full sparse array.

The radar nodes and sparse array of the distributed MIMO radar collaborative detection platform are shown in Figure 12. In Figure 13, the full sparse array was deployed over a 350 m × 150 m area. The single radar node of the system is a phased array consisting of 14 identical antenna elements arranged in a triangular mode, and the node array can adjust the array orientation within a certain range. Under the unified time-frequency benchmark of the distributed MIMO radar platform, the control center can simultaneously dispatch radar working parameters such as working frequency, sampling rate, and signal form to multiple selected nodes. In addition, the control center can also dispatch working mode orders, such as receiving only, transmitting only, or simultaneously transmitting and receiving. After receiving the echo signal, the node array element will transmit it to the designated server for processing through the high-speed optical fiber network.

#### 5.2.1. Basic Components of Distributed MIMO Radar System

**Figure 14:**
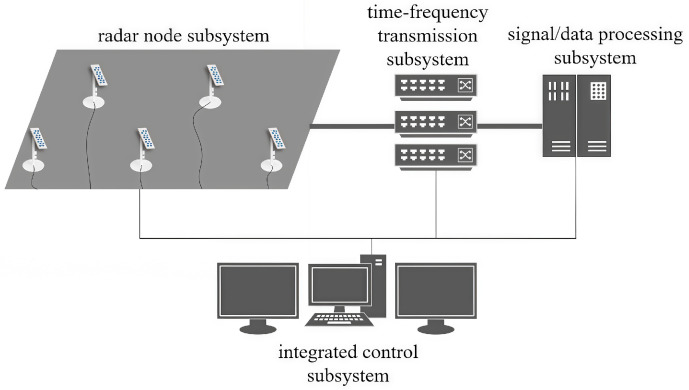
Distributed MIMO Radar System Components.

The basic components of the distributed MIMO radar test system on which this study is based consist of four subsystems, i.e., the radar node subsystem, the time-frequency transmission subsystem, the signal/data processing subsystem, or a the integrated control subsystem. The composition of these subsystems is shown in Figure 14. Specifically, a single node consists of multiple antenna elements, and each node can be configured as transceiver, a single transceiver, and single transmitting operation. The node subsystem has simple processing capabilities such as transceiver beam formation, multi-channel amplitude and phase correction, and monitoring functions. The time-frequency transmission subsystem regulates the transceiver synchronization function of the distributed MIMO radar system as well as the real-time transmission function of the echo data frames, thereby ensuring the time consistency and correctness of the multi-channel data. After the RF signal is demodulated and sampled at the node receiving end, the echo data are transmitted via optical fiber to the signal/data processing subsystem for phase parameter processing, parameter estimation, and trajectory generation.

The integrated control subsystem is the core component of the distributed MIMO radar system, which controls the parameter configuration of all subsystems and accounts for the monitoring and scheduling of hardware and software resources for each analysis. It can also output the processing results of the data processing subsystem in real time. The integrated control subsystem judges the battlefield environment by synthesizing the current information, and dynamically adjusts and promptly reconfigures different detection areas and different types of targets.

#### 5.2.2. Heterogeneous Platform for Signal/Data Processing

The signal/data processing server in this experimental verification system is equipped with a CPU + GPU heterogeneous platform composed of an Intel Core™ CPU (Intel Corporation, Santa Clara, CA, USA) and NVIDIA GeForce RTX™ series GPU processors (NVIDIA Corporation, Santa Clara, CA, USA).Each signal/data processing server is integrated into a unified system network through a switch. When data transmission is required among signal/data processing servers, the data storage server will function as a buffer, and then the signal/data processing server receiving the data will downloaded it from the data storage server. This approach is conducive to managing communication among signal/data processing servers while maximizing network resource utilization efficiency through reasonable management and control strategies.

##### Experimentation Plan and Procedure

The experimentation selects sparse antennas and RF transceiver nodes in a certain area, and the detection object is a non-cooperative civil aircraft (the range between the target and the radar is 45 km to 110 km, its flying altitude is 8 km to 12 km, with the RCS of approximately 100 m^2^). During the search and acquisition stage, each sparse node works independently, and each small point of *M* transmitting and *N* receiving can be virtualized into M×N radars. Once the target is detected by *N* radars at the same time, a certain range of the target will be searched in a refined manner to obtain more accurate position information, which can be regarded as M×N+1 virtualized radars.

Experimentation procedure:

1. Use the task planning client software to check the expected node to be used in the cluster node settings, fill in the GPU server to which the node data flows in the attribute settings, and then select the task planning to assign and select the corresponding pre-set task template to the node. Dispatch tasks, and set the task mode to real-time task after the task is successfully assigned.
2. After accomplishing the echo transmission preparation, initiate the corresponding signal processing task on the deployed server and start receiving data for real-time processing.
3. Observe the civil aircraft on the ADS-B software, accessed on 10 January 2022 wait for the aircraft to enter into the detection range of the radar, and observe the display and control software.

#### 5.2.3. Experimentation Evaluation Metrics

Performance evaluation indicator: The signal amplitude after coherent synthesis of multiple array elements in the system is expressed by the following:

(42) $$A=\sum_{m=1}^{M}\sum_{n=1}^{N}A_{mn},$$

where $A_{mn}$ denotes the amplitude of each channel.

Define the theoretical SNR of the system with full receiver coherence as follows:

(43) $$SNR_{ideal}=\frac{A^{2}}{P_{N}}=\frac{{\left|\sum_{m=1}^{M}\sum_{n=1}^{N}A_{mn}\right|}^{2}}{\sum_{m=1}^{M}\sum_{n=1}^{N}P_{n}^{mn}},$$

where $P_{n}^{mn}$ denotes the noise power of each channel, $P_{N}$ denotes the total noise power.

The theoretical SNR of the receiving fully coherent synthesis system is the ideal SNR for a distributed MIMO coherent synthesis radar system consisting of *M* transmitting array elements and *N* receiving array elements, and the equivalent average received SNR of a single array element can be defined as follows:

(44) $$\bar{SNR}=\frac{\bar{P_{S}}}{\bar{P_{N}}}=\frac{{\bar{A}}^{2}}{\bar{P_{N}}}=\frac{\sum_{m=1}^{M}\sum_{n=1}^{N}P_{S}^{mn}/MN}{\sum_{m=1}^{M}\sum_{n=1}^{N}P_{N}^{mn}}=\frac{{\left|\bar{\sum_{m=1}^{M}\sum_{n=1}^{N}A_{mn}}\right|}^{2}}{\bar{\sum_{M}\sum_{N}P_{N}^{mn}}},$$

In fact, the coherent synthesized signal power of a real system is the sum of the received signal powers of all array elements, and the noise power is the sum of the average noise powers received by all array elements, which can be described as follows:

(45) $$P_{real}^{S}=\sum_{m=1}^{M}\sum_{n=1}^{N}{\left|A_{mn}\right|}^{2},$$

(46) $$P_{real}^{N}=\sum_{m=1}^{M}\sum_{n=1}^{N}P_{mn}^{N},$$

The received noise between SNR array elements is not coherent, and the true SNR of the system is expressed by the following:

(47) $$SNR_{real}=10\log_{10}\frac{P_{real}^{S}}{P_{real}^{N}},$$

The system coherent efficiency is defined as follows:

(48) $$\eta =\frac{SNR_{real}/\bar{SNR}}{MN}=1-\frac{SNR_{real}}{SNR_{ideal}},$$

#### 5.2.4. Experimentation Results of Real-Time Processing Program

**Figure 15:**
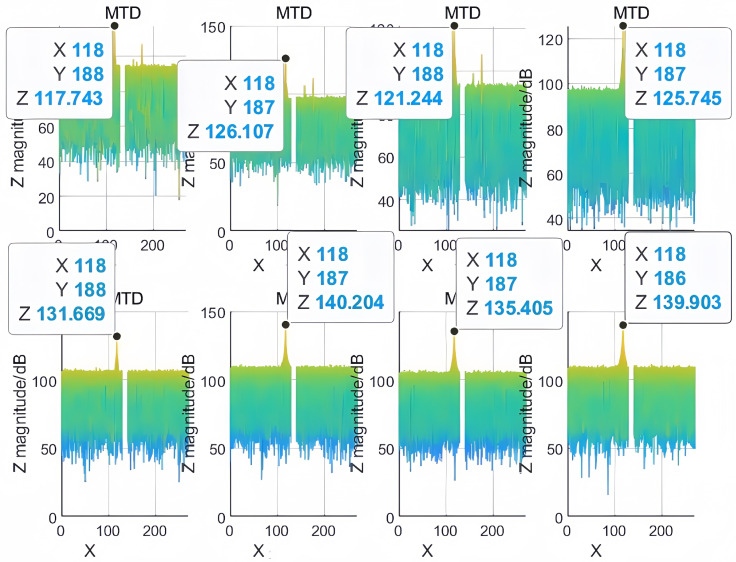
Signal amplitude of each channel.

**Figure 16:**
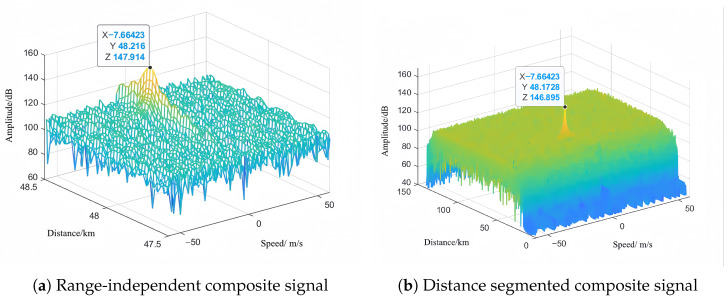
Results of synthetic signals adopting different methods.

As shown in Figure 15 and Figure 16, the synthesis efficiency of the segmented rapid matching method is approximately 61.76%, while that of the precise matching algorithm is about 69.44%. The amplitude difference after synthesis between the two algorithms approximates 1.019 dB, with the synthesis efficiency of the rapid algorithm being about 89% of that of the precise algorithm. Since the experiment did not perform transceiver calibration, the coherence performance between nodes needs further improvement. However, the operational speed of the algorithm is significantly higher than that of the precise matching algorithm.

##### Performance Analysis of GPU Acceleration

**Figure 17:**
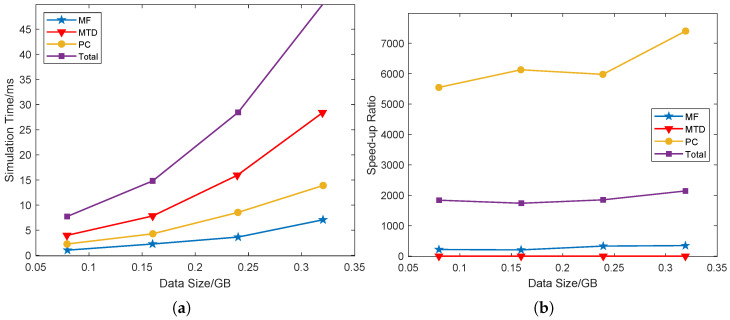
Acceleration effect between CPU and GPU (**a**) simulation time (**b**) speed-up ratio.

The signal processing of near-field distributed radar mainly consists of three parts: MF, MTD, and phase compensation (PC). To evaluate the calculating speed of these three steps, four different sizes of simulated data are used. Here, 10 Monte Carlo experiments are performed on each data set to calculate the average speed, and the results are listed in Figure 17.

From Figure 17a, it is shown that the time cost heavily depends on the data size. As shown in Figure 17b, the speedup ratio tends to increase as the data size increases. In addition, the higher the algorithm complexity is, the more speed-up potential the GPU achieves. When the data size is about 0.31GB, the GPU-based processing time is about two thousand times faster than that based on the CPU [55]. This experimental validation demonstrates the real-time capability and system integration of the proposed processing architecture, which is equally critical for near-field operation.

## 6. Conclusions

In this study, a multidimensional domain matching signal-level coherent and pulse synthesis method via GPU is proposed for near-field distributed radar detection. Two improvements of the proposed method can be summarized as follows. First, the energy of the target can be greatly accumulated by an accurate near-field phase compensation method. Second, the computation speed is significantly improved via a parallel algorithm designed in GPU devices. Third, we investigated the rationale of near-field detection, as well as the FD-LFM waveform and its signal processing, proposing a near-field multidimensional matching scheme based on FD-LFM.

Simulation experiments were designed for the proposed algorithm. The experimental results have verified that our proposed algorithm is capable of realizing high-precision near-field broadband multidimensional matching within a short time frame. The results also indicate that compared with the conventional CPU-based method, the GPU-based method improves the near-field processing efficiency by two thousand times. This effectively solves the problem of near-field signal processing in distributed MIMO radar systems, offering new perspectives for further research in related fields.

## Figures and Tables

**Table 1:** FD-LFM MIMO radar simulation parameters.

Radar Parameters	Numerical Value	Unit of Measurement
Transmit nodes	4	Unit
Receive nodes	32	Unit
Transmit carrier frequency	1.5	GHz
Pulse Repetition Period	500	us
Pulse width	50	us
Signal bandwidth	1	MHz
Stepped frequency	1	MHz
Sampling frequency	10	MHz
CPI pulse number	32	Unit

**Table 2:** Multi-targets simulation parameters.

Target Parameters	Target 1	Target 2	Target 3
Range	3.5 km	5.0 km	6.5 km
Azimuth	60°	60°	61°
Speed	31 m/s	30 m/s	30 m/s
SNR	−20 dB	−20 dB	−20 dB

## Data Availability

The original contributions presented in this study are included in the article. Further inquiries can be directed to the corresponding author.

## Appendix A. Frequency–Domain Representation of the Transmitting Signal at the Transmitting End

After conducting FFT with respect to the transmitted signal, we obtain

(A1) $$S_{m}^{\left(e\right)}\left(f\right)=\int_{-\infty }^{\infty }\mathrm{rect}\left(\frac{t}{T_{p}}\right)e^{j\pi \mu t^{2}}e^{-j2\pi ft}dt,$$

By using the stationary phase point principle and Fresnel integration, the frequency–domain derivation of the *m*-th transmitted signal is expressed as follows.

The phase with time is as follows:

(A2) $$\varphi \left(t\right)=-2\pi ft+\pi \mu t^{2},$$

the derivative of phase time *t* is calculated by the following:

(A3) $$\frac{d\varphi (t)}{dt}=-2\pi f+2\pi \mu t,$$

From the stationary phase point, the point where the phase changes slowest, is the point where the derivative is 0.

Let $\frac{d\varphi (t)}{dt}=0$, then $t^{*}=\frac{f}{\mu }$, we obtain the following:

(A4) $$\begin{array}{cc} S_{m}^{\left(e\right)}\left(f\right) & =\mathrm{rect}\left(\frac{f}{B}\right)e^{j\pi \mu {\left(\frac{f}{\mu }\right)}^{2}}e^{-j2\pi f\left(\frac{f}{\mu }\right)}e^{j\frac{\pi }{4}}\\ & =\mathrm{rect}\left(\frac{f}{B}\right)e^{-j\pi \frac{f^{2}}{\mu }}e^{j\frac{\pi }{4}} \end{array}.$$
